# German Treatment of Croup and Diptheria

**Published:** 1879-02

**Authors:** 


German Treatment of Croup and Diphthrria.—The following treatment of croup and diphtheria is advocated by Dr. Taube, in the *Deutsche Zeitschrift fur Prakt. Medicin*, September, 1878. An ordinary inhaler is to be taken, full of water, into which is to be put each time it is used fifteen drops of the oil of turpentine. The child should be wrapped in a sheet and placed on the mother's lap, while another person holds the apparatus for eight or ten minutes, about three inches from its mouth. It is as well to grease the child's face and to protect the eyes with a cloth. At first the inhalation should be practiced every hour, day and night, the favorable result of which will soon be apparent. With regard to the carbolic acid injection, it should be made into the submucous tissue, half the contents of an ordinary hypodermic syringe, containing a weak solution of the acid (3 to 1000) being injected two or three times a day into the tonsils — subject to certain modifications, as the case may require. Taube would recommend the following plan: 1. Hourly inhalations of the oil of turpentine throughout the day and night. 2. Injection of carbolic acid two or three times a day. 3. From one to two teaspoonfuls of Port wine of Madeira, to be given every hour; cold compress to the neck; two or three times a day a warm bath with the cold affusion; and small doses of the infusion of digitalis, to which a little benzonic acid is added. The nourishment should consist of eggs and milk only. Constipation is to be met with linseed oil, and for the separation of the false membrane, sulphate of copper, or tracheotomy, with turpentine inhalations through and above the canula.—*Med. and Surg. Reporter.*

